# A novel role of bone morphogenetic protein 6 (BMP6) in glucose homeostasis

**DOI:** 10.1007/s00592-018-1265-1

**Published:** 2018-12-11

**Authors:** Martina Pauk, Tatjana Bordukalo-Niksic, Jelena Brkljacic, Vishwas M. Paralkar, Amy L. Brault, Ivo Dumic-Cule, Fran Borovecki, Lovorka Grgurevic, Slobodan Vukicevic

**Affiliations:** 1 Laboratory of Mineralized Tissues, Center for Translational and Clinical Research, School of Medicine, University of Zagreb, Salata 11, Zagreb, Croatia; 2 Pfizer Inc, Groton, CT USA; 3 Department for Functional Genomics, Center for Translational and Clinical Research, School of Medicine, University of Zagreb, Salata 2, Zagreb, Croatia

**Keywords:** Bone morphogenetic protein 6 (BMP6), Glucose, Insulin, *Ob*/*ob* mice, PepCK

## Abstract

**Aims:**

Bone morphogenetic proteins (BMPs) are involved in the development and homeostasis of multiple organs and tissues. There has been a significant focus on understanding the role of BMPs in pancreatic β-cell dysfunction associated with type 2 diabetes (T2D). Our objective was to investigate the relationship between BMP6 and glucose homeostasis.

**Methods:**

*Ob*/*ob* mice were treated with BMP6 for 6 days and analyzed for insulin release, body weight, lipid parameters and glucose tolerance. Quantitative real-time PCR, chromatin immunoprecipitation and glucose output assays were used to assess BMP6 effect on gluconeogenesis in rat hepatoma H4IIE cells. Specificity of BMP6 receptors was characterized by the utilization of various receptor Fc fusion proteins in luciferase reporter gene and glucose output assays in INS1 and H4IIE cells.

**Results:**

Treatment of *ob*/*ob* mice with BMP6 for 6 days resulted in a reduction of circulating glucose and lipid levels, followed by a significantly elevated plasma insulin level in a dose-dependent manner. In addition, BMP6 improved the glucose excursion during an oral glucose tolerance test, lowering the total glycemic response by 21%. In rat H4IIE hepatoma cells, BMP6 inhibited gluconeogenesis and glucose output via downregulation the PepCK expression. Moreover, BMP6 inhibited glucose production regardless of the presence of cAMP, antagonizing its glycogenolytic effect. BMP6 acted on pancreatic and liver cells utilizing Alk3, Alk6 and ActRIIA serine/threonine kinase receptors.

**Conclusions:**

Collectively, we demonstrate that BMP6 improves glycaemia in T2D mice and regulates glucose metabolism in hepatocytes representing an exciting prospect for future treatments of diabetes.

## Introduction

Bone morphogenetic proteins (BMPs) are members of the transforming growth factor-β (TGF-β) superfamily [1, 2]. BMPs signal to the responding cells via hetero-oligomeric complexes of BMP type I (ACVR1A (Alk2), BMPR1A (Alk3), and BMPR1B (Alk6)) and type II (BMPR2, ActRIIA, and ActRIIB) serine/threonine kinase receptors [3]. Although, BMPs were originally discovered by their ability to induce bone and cartilage formation, they play distinct roles in the development of other organ and tissue systems [3, 4].

Several studies have indicated an important role of BMPs in the development of pancreas [5]. However, little is known about a potential role of BMPs in the regulation of glucose homeostasis and insulin resistance. So far, BMP9 has been identified as a hepatic factor that regulates blood glucose via inhibiting hepatic glucose production and lowering glycaemia levels in normal and diabetic mice [6]. Involvement of BMPs in glucose metabolism has also been demonstrated in mice with attenuated BMPR1A signaling in β-cells, which lead to decreased expression of key genes involved in insulin gene expression, glucose sensing and diabetes due to impaired insulin secretion [7]. In addition, systemic administration of BMP4 to adult mice stimulated insulin secretion [7], while BMP7 stimulated differentiation of pancreatic exocrine tissue into functional islet insulin-producing cells [8] and restored glucose uptake in cells with attenuated insulin signaling [9]. Also, BMP2 and 6 were proposed as new potent insulin sensitizers in adipocytes based on their ability to increase glucose uptake and to regulate genes involved in glucose and fatty acid metabolisms [10]. These results suggested that BMPs have essential roles in pancreas organogenesis and endocrine cell differentiation and are involved in regulating glucose metabolism in liver, pancreas, adipose tissue and muscle. However, there is no evidence of the bioavailability of BMPs and tissue-specific function of BMPs in diabetic animal models.

It has been previously reported that mis-expression of BMP6 leads to agenesis of the pancreas and reduction in the size of the stomach and the spleen, causing fusion of the liver and duodenum [11]. Taken together, there is an emerging need to understand the precise role of BMP6 in the liver and pancreas in maintaining glucose homeostasis. The present study aimed to further explore the beneficial effect of BMP6 on glucose metabolism and insulin secretion utilizing rodent models of T2D.

## Materials and methods

### Animals

Animal care was in compliance with SOPs of Animal facility; the European convention for the protection of vertebrate animals used for experimental and other scientific purposes (ETS 123). All experiments were approved by the Institutional Animal Care Review and Ethics Committee, University of Zagreb, School of Medicine. Eight-week-old male *ob*/*ob* mice (Jackson Laboratory) were housed in conventional laboratory conditions with standard GLP diet (Mucedola, Italy) and fresh water ad libitum. Mice were treated with BMP6 for 6 days (once every 48 h; total of 5 BMP6 i.v. injections) at 10, 40 and 60 µg/kg, while control group received a vehicle (PBS) (*n* = 8–10 per group). Following an overnight fast, on day 7, 2-h post-rhBMP6 dose, OGTT was performed and blood glucose levels were monitored at 15, 30, 60 and 120 min. Glucose was measured using an ACCU-CHECK® glucose assay (Roche). At day 6, 2-h post-rhBMP6 dose, serum insulin levels were measured using Insulin ELISA kit (Mercodia). Total cholesterol and triglyceride were measured by enzymatic methods using kits (Roche) and Cobas 6000 analyzer (Roche).

### Recombinant human BMP6

The manufacturing of rhBMP6 was conducted by Genera Research (Kalinovica, Croatia). Engineered Chinese Hamster Ovary (CHO) cell line was used to produce and purify rhBMP6 from the media using heparin affinity and hydrophobic interaction chromatography, followed by the reverse-phase HPLC. Protein was lyophilized and stored at − 20 °C in vials containing 0.5 mg > 99% pure rhBMP6.

### Glucose output assay

Rat hepatoma H4IIE cells (1 × 10^5^ per well in 96-well plate) were cultured in DMEM with low glucose (Gibco). After 6 h, media was replaced with glucose production media (Gibco), 20 mM sodium lactate and 2 mM sodium pyruvate. After 18 h, treatments were applied in fresh glucose production media. In BMP6 experiments, cells were treated with increasing BMP6 concentrations for 24 h. In cAMP experiments, cells were treated for 12 and 24 h with 300 ng/mL BMP6 or in combination with 0.5 mM Bt_2_cAMP. In siRNA ActRIIA experiments, cells were treated for 24 h with BMP6 at 1, 10 and 100 ng/mL. In Fc experiments, cells were treated for 24 h with 100 ng/mL rhBMP6, and rhAlk3 Fc, rhAlk6 Fc and rhActRIIa Fc chimera proteins (R&D Systems) at 0.1, 1, 10 and 50 µg/mL. In all experiments, DMEM served as a control. Media were collected at indicated times and assayed using Amplex Red Glucose Assay (Invitrogen).

### Chromatin immunoprecipitation

H4IIE cells were maintained in DMEM (Invitrogen) and harvested untreated or 3 and 7 h following rhBMP6 (100 ng/mL) application. The standard ChIP protocol was performed as described [12], except of using antibodies against Smad1,2,3,5,8 (Santa Cruz Biotechnology), RNA polymerase II (Santa Cruz Biotechnology) and histone H3 acetylated at lysines 9 and 14 (Diagenode). The original immunoprecipitated and total input DNA aliquots were used to assess the quality of the ChIP. The Smad occupancy was detected by qPCR on a Roche LightCycler at the insulin and *PepCK* promoters. The quality of the ChIP was calculated as fold enrichment over myoglobin exon 2 non-binding control region.

### Gene expression analysis

After treatment of H4IIE cells with 100 ng/mL BMP6 for 12 h, one-step quantitative RT-PCR was performed on 1 µg of total RNA using superscript first-strand synthesis system (Invitrogen) with random hexamer primers according to the manufacturer’s protocol. Gene expressions were measured using LightCycler FastStart DNA Master SYBR Green kit (Roche) in the LightCycler instrument (Roche) as described [13]. Results were represented as fold change of comparative vehicle expression level, following normalization to hypoxanthine phosphoribosyltransferase 1 (HPRT1).

### Luciferase reporter gene assay in INS1 cells

INS1 cells (2.5 × 10^4^ per well in 48-well plate) were transfected with a reporter plasmid consisting of BRE from the *Id-1* promoter fused to a luciferase reporter gene. After 24 h, the medium was changed to serum-free DMEM/F-12 for 7 h. Cells were then treated for 17 h with serum-free DMEM/F-12 (Control), rhBMP6 (20 ng/mL) and increasing concentrations of Alk3 and Alk6 chimera proteins, and luciferase activity was measured using the Promega luciferase assay reagent.

### Gene knockdown of ActRIIA in H4IIE cells

H4IIE cells (2 × 10^6^ per 10-cm^2^ dish) were transfected with ActRIIa siRNA (Thermo Scientific). In brief, 6 mL of OptiMEM media (Gibco) containing 6 µL of DharmaFect 1 transfection reagent (Thermo Scientific) and 100 nM of siRNA was added to the dish and incubated overnight. The following day, the transfection media was removed and replaced with fresh growth media. After 24 h, the cells were trypsinized and plated in a 96-well plate and a glucose output assay was performed.

### Data analyses

The data were expressed as mean ± standard error of mean (SEM) or standard deviation (STDEV). Changes in gene expression and serum parameters were evaluated using the two-tailed Student *t* test. The results were considered significant when *p* < 0.05.

## Results

### BMP6 improves glucose tolerance in *ob*/*ob* mice

**Fig. 1:**
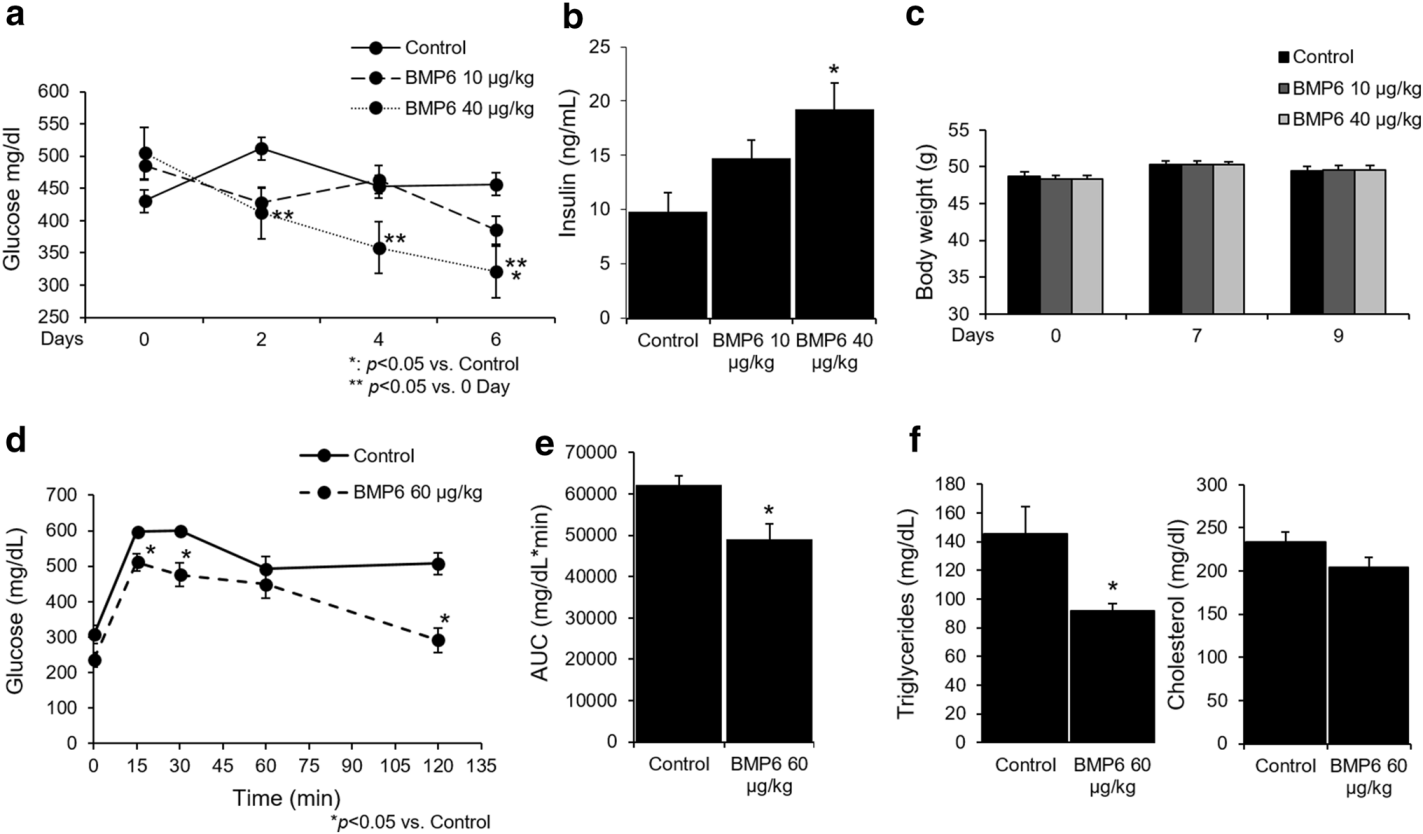
BMP6 is a hypoglycemic agent in *ob*/*ob* mice. Following BMP6 therapy (10 and 40 µg/kg; every 48 h for 6 days) in 8-week-old *ob*/*ob* mice (*n* = 8–10), we measured at 2-h post-dose **a** fed plasma glucose every other day, **b** circulating insulin levels at day 6, and **c** body weights during 9 days of treatment. Oral glucose tolerance test (OGTT) was performed on day 7, 2-h post-BMP6 (60 µg/kg) treatment and **d** glucose was measured at 0, 15, 30, 60 and 120 min and **e** AUC was calculated. Upon BMP6 (60 µg/kg) treatment for 6 days, **f** circulating cholesterol and triglycerides levels were measured. Results are reported as the mean ± SEM. *p < 0.05 vs. control, **p < 0.05 vs. 0 day

We tested the ability of BMP6 treatment to influence the plasma glucose levels in *ob*/*ob* mouse model. First, we assessed the ability of lower BMP6 doses (10 and 40 µg/kg; every 48 h) following 6 days of therapy on maintaining glucose levels. BMP6 at 40 µg/kg was efficient in significantly lowering fed plasma glucose from second day onward (Fig. 1a). Importantly, plasma insulin levels measured at day 6 of BMP6 treatment were significantly elevated relative to control in a dose–response manner (Fig. 1b). We did not observe any change in body weight upon BMP6 administration through therapy (Fig. 1c). Treatment with BMP6 at 60 µg/kg following 6 days demonstrated hypoglycemic effect by decreasing plasma glucose at 236.2 mg/dL compared to 307.3 mg/dL of the control. On day 7, 2-h post-dose, OGTT was performed, and BMP6 significantly lowered plasma glucose levels and improved glucose tolerance compared to control (Fig. 1d). Total glycemic response was 21% lower following BMP6 treatment (area under the curve (AUC), *p* = 0.0128) (Fig. 1e). Since increased plasma levels of triglyceride and cholesterol are frequently associated with insulin resistance, we wanted to determine their levels following 6 days of BMP6 treatment. While BMP6 had no effect on cholesterol, plasma triglycerides levels were significantly decreased (Fig. 1f). Altogether, these results indicate BMP6 effect on maintaining plasma glucose via affecting the insulin release in *ob*/*ob* mice.

### BMP6 regulates glucose homeostasis via regulation of hepatic glucose output

**Fig. 2:**
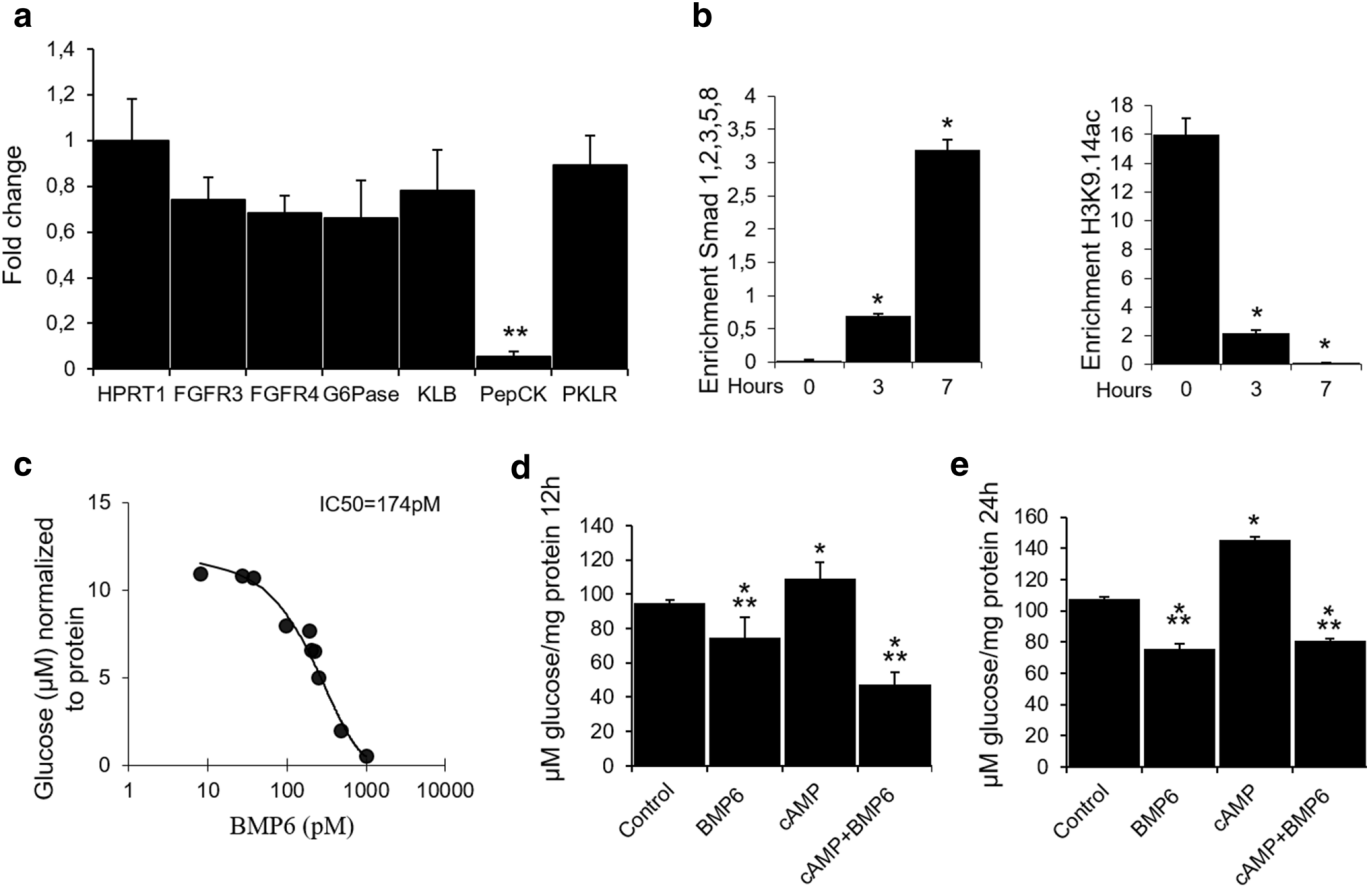
Role of BMP6 in hepatic gluconeogenesis. Following BMP6 (100 ng/mL) treatment in H4IIE cells (*n* = 4) at 12 h, we measured **a** gene expression by qRT-PCR of fibroblast growth factor 3 (FGF3), fibroblast growth factor receptor 4 (FGFR4), glucose-6-phosphatase (G6Pase), betaKlotho (KLB), phosphoenolpyruvate carboxykinase (PepCK) and pyruvate kinase L/R (PKLR) and hypoxanthine phosphoribosyltransferase 1 (HPRT1). Results are represented as fold change of vehicle expression level, following normalization to HPRT1. **b** PepCK promoter recruitment was assessed 3 and 7 h following BMP6 (100 ng/mL) treatment by chromatin immunoprecipitation using antibodies against Smad1,2,3,5 and 8 or histone H3 (*n* = 2). Glucose output (*n* = 4) was measured following treatment with **c** increasing BMP6 concentrations at 24 h, and BMP6 (300 ng/mL) and cAMP (0.5 mM) at **d** 12 and **e** 24 h. Results are reported as the mean ± STDEV. *p < 0.05, **p < 0.01 vs. control

To understand the mechanism of BMP6 action, we studied its effect in H4IIE rat hepatoma cell line. BMP6 treatment significantly downregulated PepCK expression (95%) with very little change in the expression of other metabolic enzymes at 12-h treatment (Fig. 2a). To ascertain possible alterations in promoter recruitment of genes related to the insulin pathway, chromatin immunoprecipitation (ChIP) experiments on H4IIE cells were performed. Using the ChIP-grade antibody against Smad 1,2,3,5 and 8, we observed an increased PepCK promoter recruitment 7 h following BMP6 treatment (Fig. 2b). The same was not observed with specific primers for the insulin promoter. To ensure that potential observed binding events are the result of an active transcription, we performed ChIP experiments using antibodies specific for RNA Polymerase II and histone H3 acetylated at lysine 9 and 14. We showed a marked decrease in PepCK promoter recruitment by acetylated H3 histones following BMP6 administration (Fig. 2b), while no recruitment of the insulin promoter was observed. The RNA polymerase II binding to the insulin and PepCK promoters was not detected. These results point to the possible negative regulation of PepCK transcriptional activity by BMP6. Next, we analyzed BMP6 effect on glucose output in H4IIE cells and showed that BMP6 significantly inhibited hepatic glucose output in a dose–response manner with an IC-50 of 174 pM (Fig. 2c). As one of the key enzymes in hepatic gluconeogenesis, PepCK is regulated mainly by glucagon and insulin that stimulate and repress its gene transcription, respectively. Since glucagon stimulation on PepCK is exerted via cyclic adenosine monophosphate (cAMP), we tested whether BMP6 may act to antagonize its glycogenolytic effect. BMP6 significantly decreased the glucose output 12 and 24 h following treatment, while cAMP addition was followed by a higher glucose output (Fig. 2d, e). Inhibitory effect of BMP6 on glucose output was sustained even after stimulation with cAMP. Overall, BMP6 directly decreased the hepatic glucose production through downregulation of the PepCK expression regardless of the presence of cAMP.

### Characterization of BMP6 receptors in mediating glucose output

**Fig. 3:**
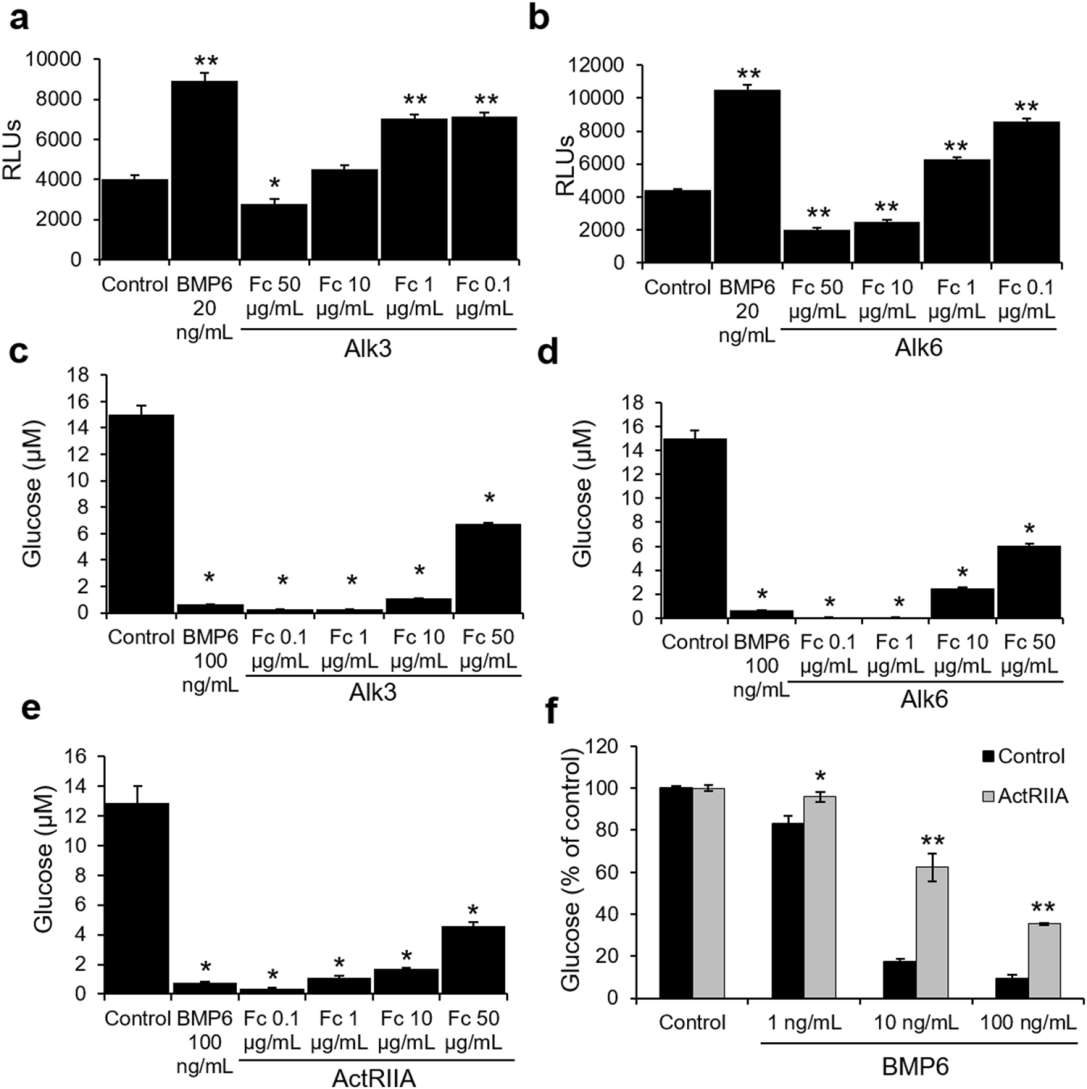
Characterization of BMP6 receptor in mediating glucose output. INS1 cells (*n* = 5) transfected with BRE-Luc were treated for 17 h with BMP6 (20 ng/mL) and **a** Alk3 and **b** Alk6 chimera proteins at 0.1, 1, 10 and 50 µg/mL. Luciferase activity was measured and expressed as relative light units. H4IIE cells (*n* = 2) were treated for 24 h with **c** Alk3, **d** Alk6 and **e** ActRIIA Fc at 0.1, 1, 10 and 50 µg/mL, and glucose was measured in the media. **f** H4IIE cells (*n* = 2) were silenced for ActRIIA expression, treated with BMP6 (1, 10 and 100 ng/mL) and glucose was measured in the media. Results are reported as the mean ± STDEV. *p < 0.05, **p < 0.01 vs. control and no treatment

The specificity of BMP6 actions in vitro was further characterized by the utilization of various receptor Fc fusion proteins. These studies identified that Alk3 and Alk6 (BMPR1A and 1B) were able to inhibit BMP6-stimulated luciferase activity of a BRE promoter construct in INS1 cells (Fig. 3a, b). In H4IIE, Alk3 and Alk6 Fc fusions partially inhibited BMP6 effects on the glucose output, whereas ActRIIA Fc fusion was partially able to rescue the inhibition of glucose output by BMP6 (Fig. 3c, d, e). Knockdown of the ActRIIA gene expression reversed the inhibition of glucose output by BMP6 indicating that in hepatocytes ActRIIA plays a major role in transducing BMP6 activity (Fig. 3f). Together, these data indicate that BMP6 has a direct specific action on pancreatic cells and hepatocytes, and utilizes Alk3, Alk6 and ActRIIA to initialize signaling events that ultimately result in gene transcription regulation.

## Discussion

There has been a significant focus on understanding the involvement of BMPs in β-cell dysfunction associated with T2D, but there are many conflicting results regarding the effect of BMPs on glucose homeostasis and pancreatic cell function. Here, we tested whether BMP6 has beneficial effect on glucose homeostasis and insulin secretion using T2D mice model. In *ob*/*ob* mice, BMP6 was effective in lowering plasma glucose levels during 6 days of therapy, but more importantly, it improved glucose excursion during an OGTT. Also, BMP6 significantly elevated serum insulin levels in a dose–response manner. Previously, BMP9 was shown to be effective in lowering plasma glucose at a high dose of 5 mg/kg and exerted its effect mainly via metabolism in the liver [6]. In comparison, BMP6 at doses of 10–60 µg/kg i.v. reduced glycaemia in diabetic mice with a prolonged effect on maintaining glucose levels. Unlike insulin which acts rapidly in a hormone-like manner, BMP6 has a delayed effect of several hours to several days, respectively, in lowering the plasma glucose. Therefore, it seems likely that BMP6 affects insulin-dependent pathway, ruling out a possibility that it functions as insulin mimetic and/or sensitizer. Similar effect of BMP6 on glucose metabolism was also obtained in our laboratory on other rodent models without obvious alterations in leptin, which implicates that BMP6 affects glucose metabolism independently on possible changes in leptin metabolism (data not shown).

Since liver has a major role in the control of glucose homeostasis, we addressed the question if the acute lowering of glucose was caused by an effect of BMP6 on glucose metabolism in the liver. In experiments on H4IIE cells, BMP6 treatment significantly reduced PepCK expression and there was a marked decrease in PepCK promoter recruitment following BMP6 administration. PepCK plays a major contributory role in the hyperglycemia characteristic of diabetes, as it catalyzes the reversible decarboxylation of oxaloacetate to yield phosphoenolpyruvate and CO_2_. Therefore, it was of our interest to further characterize BMP6 and cAMP action in these cells, since cAMP is known to play a major role in the stimulation of PepCK gene transcription. Our results showed that BMP6 directly decreased the glucose production via downregulating PepCK expression at 12 and 24 h treatment in these cells independently of cAMP. Altogether these results indicate that BMP6, may also act through inhibiting gluconeogenesis in the liver.

BMP receptors and Smad proteins are expressed in β-cells, whereas BMP ligands are present at low levels, suggesting that the BMP signal transduction is present in the pancreatic islets and that external sources of BMPs are important activators of BMP signaling in the pancreas [5, 7, 14]. BMP6 preferentially binds and signals through Alk2, but can also utilize Alk3 to elicit the Smad signaling [15, 16]. Interestingly, Alk3 deletion leads to diabetes due to impaired insulin secretion and to a decreased expression of genes involved in insulin gene expression [7]. We found that BMP6 stimulates Smad signaling through binding to Alk3, Alk6 and ActRIIA serine/threonine kinase receptors in H4IIE and β-cells, initializing signaling events that result in gene transcription regulation.

The presence of BMPs in local microenvironment is important throughout development and homeostasis of almost all organs. BMP6 is required for systemic skeletal development and homeostasis during adult life. We have previously shown that systemic application of BMP6 restores the bone inductive capacity, micro-architecture, and quality of the skeleton in osteoporotic rats [17]. BMP6 exerts its powerful effects on bone volume (BV) by promoting differentiation of MSCs to osteoblasts and decreasing differentiation of hematopoietic stem cells towards the osteoclasts, uncoupling bone remodeling by promoting bone formation and reducing bone resorption. No known therapeutic agent achieves both effects by in vivo systemic administration [18]. Diabetes is associated with changes in bone formation and stem cell differentiation resulting in altered bone mineral density (BMD) and bone structure [19]. In addition, both types of diabetes lead to reduced bone strength and increased risk of skeletal fractures. Also, many reports on diabetes show increased osteoclastogenesis and decreased osteoblast formation due to preferential differentiation of mesenchymal stem cells in the marrow towards adipocytes [20, 21]. This could be attributed at least in part by reduced expression of factors that stimulate osteoblasts such as BMPs as was previously reported in rats with T2D [22]. Thus, the role of BMP6 in bone and pancreas development and homeostasis, as well as mediation of long-term effects of bone on glucose metabolism supports its unique properties in systems biology. Furthermore, its resistance to noggin and systemic availability separates BMP6 from other TGFβ superfamily members [23].

We demonstrated that BMP6 has a delayed effect in lowering plasma glucose in diabetic mice, unlike insulin which acts rapidly in a hormone-like manner. The mechanism is still not fully understood, but at least part of the mechanism seems to involve insulin release and inhibition of gluconeogenesis. Nevertheless, this novel role of BMP6 in the pancreas, liver and the systemic glucose homeostasis suggests a unique function of BMP6 that can be utilized for the treatment of diabetes.

## Abbreviations

BMP: Bone morphogenetic protein
OGTT: Oral glucose tolerance test
TGF-β: Transforming growth factor-β
ACVR1A: Activin A receptor, type I
BMPR: Bone morphogenetic protein receptor
ALK: Activin receptor-like kinase
ActRIIA: Activin receptor type IIA
DMEM: Dulbecco’s modified eagle medium
ChIP: Chromatin immunoprecipitation
AUC: Area under the curve
PepCK: Phosphoenolpyruvate carboxykinase
cAMP: Cyclic adenosine monophosphate
RT-PCR: Real-time polymerase chain reaction
T2D: Type 2 diabetes
